# Pose Estimation with a Kinect for Ergonomic Studies: Evaluation of the Accuracy Using a Virtual Mannequin

**DOI:** 10.3390/s150101785

**Published:** 2015-01-15

**Authors:** Pierre Plantard, Edouard Auvinet, Anne-Sophie Le Pierres, Franck Multon

**Affiliations:** 1 M2S Laboratory, University Rennes 2, ENS Rennes, Avenue Robert Schuman, 35170 Bruz, France; E-Mail: Franck.Multon@irisa.fr; 2 FAURECIA Automotive Seating, ZI de Brières les Scellés, B.P. 89 91152 Etampes, France; E-Mail: anne-sophie.lepierres@faurecia.com; 3 Ecole Polytechnique de Montréal, C.P. 6079, Succursale Centre-ville, Montréal, H3C 3A7 QC, Canada; E-Mail: edouard.auvinet@polymtl.ca; 4 CHU Sainte Justine de Montréal, 3175, Chemin de la Côte-Sainte-Catherine, Montréal, H3T1C5 QC, Canada; 5 INRIA, MimeTIC team, Campus Universitaire de Beaulieu, 35042 Rennes, France

**Keywords:** Kinect, accuracy, virtual mannequin

## Abstract

Analyzing human poses with a Kinect is a promising method to evaluate potentials risks of musculoskeletal disorders at workstations. In ecological situations, complex 3D poses and constraints imposed by the environment make it difficult to obtain reliable kinematic information. Thus, being able to predict the potential accuracy of the measurement for such complex 3D poses and sensor placements is challenging in classical experimental setups. To tackle this problem, we propose a new evaluation method based on a virtual mannequin. In this study, we apply this method to the evaluation of joint positions (shoulder, elbow, and wrist), joint angles (shoulder and elbow), and the corresponding RULA (a popular ergonomics assessment grid) upper-limb score for a large set of poses and sensor placements. Thanks to this evaluation method, more than 500,000 configurations have been automatically tested, which would be almost impossible to evaluate with classical protocols. The results show that the kinematic information obtained by the Kinect software is generally accurate enough to fill in ergonomic assessment grids. However inaccuracy strongly increases for some specific poses and sensor positions. Using this evaluation method enabled us to report configurations that could lead to these high inaccuracies. As a supplementary material, we provide a software tool to help designers to evaluate the expected accuracy of this sensor for a set of upper-limb configurations. Results obtained with the virtual mannequin are in accordance with those obtained from a real subject for a limited set of poses and sensor placements.

## 1. Introduction

Analyzing human postures and movements at workstations is a key issue in order to evaluate potentials risks of musculoskeletal disorders. To this end, methods have been developed to evaluate exposure to risk factors in the workplace. They can be divided into three groups according to the accuracy of the data collection and the measurement technique that is used: self-report, observational methods, and direct measurement [1].

The first group, self-report methods, can take many various forms such as rating scales, questionnaires, checklists, or interviews. This kind of approach focuses on the assessment of physical workload, body discomfort, or work stress, which are difficult to objectively measure. Therefore, although this method is easy-to-use, it is not reliable enough and could result in misleading interpretations [2,3].

The second group, observational methods, consist of direct observation of the worker and his tasks. These are widely used in industry. One of the most well-known methods is the Rapid Upper Limb Assessment (RULA) [4]. This kind of tool requires to rate the pose of the worker, generally based on an estimation of joint angles. These methods are easy-to-use, generally do not require complex setups, and can be used to evaluate a wide range of working tasks [5]. However, since data collection is obtained through subjective observation or simple estimation of projected angles in videos/pictures, the method would be subject to inaccuracy or bias across different observers [6]. Video-based systems have been introduced to partly overcome these limitations, especially to increase accuracy and robustness of joint angle estimation. They do not restrict or disturb the natural motions of the workers [7], but it remains difficult and tedious to obtain 3D information and to place cameras at suitable/unobstructed positions in congested workplaces.

Thirdly, direct methods, unlike the others, collect data directly from sensors attached to the worker's body. They are preferred in a research context, but are difficult to implement in real work situations [1]. Posture assessment with goniometric devices are widely used in ergonomics and provide high accuracy for epidemiologic studies [8]. However, wearing these devices may cause discomfort and affect postural behavior. Moreover, this tool is limited to planar movements, and will become problematic for complex joints, such as the shoulder. Magnetic systems are also used for motion tracking [9]. They can continuously measure joint motion in six degrees of freedom, but are difficult to use on site because of perturbations of the magnetic fields due to machines and ferromagnetic objects. Finally, inertial sensors (such as accelerometers or gyroscope sensors) can accurately assess human pose [10,11]. However, these devices can be disturbed by the environment conditions of the real work situations (such as vibrations). All these wearing sensors are sensitive to placement on the body, making their use in the workplace difficult.

Advances in the field of markerless motion capture can potentially offer an opportunity to overcome these limitations of disturbing the natural motion of workers. Recent development of low-cost depth cameras such as the Microsoft^®^ Kinect sensor provides easy-to-use, markerless, calibration-free and cheap alternatives. This device (based on PrimeSense Technology, Tel Aviv, Israel) [12–14] is composed of an infrared projector of structured light and an infrared camera that returns a depth image of the scene at 30 Hz. The associated software enables us to identify pixels that belong to the body parts by the use of a randomized decision forest algorithm. This body part classification finally leads to the estimation of twenty 3D joint positions of the main joints in the human body [15].

Several authors have studied the accuracy of the kinematic data provided by the Kinect in various application domains [16–19]. The hardware accuracy has been investigated through the study of the depth map [20–22]. The results show that the hardware component of the Kinect is accurate enough to capture 3D objects in a workplace environment [20]. The accuracy and reliability of the kinematic data provided by the Shotton's algorithm have also been investigated, especially in rehabilitation. The joint positions [23,24] and the resulting joint angle errors [16,18,24,25] look promising for most clinical uses. However, only a few postures were studied in previous works, mostly in clinical analysis for very limited and standardized postures performed in 2D anatomical plans. Although the Kinect looks promising for capturing 3D data of work activities over periods of time, it raises new questions that have not been addressed yet. While several Kinect-based applications were developed to improve the postural assessment of observational methods [26–28], the reliability of the angular joint values delivered by the systems have not been accurately evaluated in ergonomic applications yet. Indeed, the workplace environment is often crowded with many potential occlusions that block a video-based system from seeing the subject's body properly, leading to potential critical errors [29]. Moreover, placing the Kinect at an ideal position, *i.e.*, 2 m away from the subject at a convenient height, is usually impossible. According to the Kinect recommendations (NUI Kinect for Windows 1.8) the accuracy of the sensor decreases when these ideal conditions are not satisfied. Thus, it also seems necessary to determine the influence of the Kinect view angle relative to the worker, as suggested in [26].

Evaluating a wide range of poses and sensor configurations is a difficult task, especially if we need to consider several repetitions for each condition. To address this problem, [30] suggests using simulation with an anthropometric 3D mesh. They proposed to deform the anthropometric 3D mesh to mimic the desired poses and simulate the resulting depth image of the scene, as a Kinect would do under the same conditions. Using a simulated depth map representing a 3D mesh instead of real humans before running the Shotton's algorithm enables us to test a large set of configurations and repetitions, as Shotton did in [15]. It also enables us to test the kinematic data reconstruction algorithm independently from the hardware device. The quality of the depth images measured with a Kinect has been extensively evaluated in previous works [20–22] and the new version of the Kinect offers better quality. However the accuracy of the kinematic reconstruction software is less clear whatever the quality of the sensor is, especially for complex 3D poses in constrained environments, such as the workplaces. Using rendered virtual mannequins as an input of this kinematic reconstruction software will enable us to clarify the reliability of this estimation in realistic conditions, independently from the sensor's accuracy.

In this work, we consequently propose to evaluate the accuracy of kinematic data computed by the Kinect software according to simulated depth images associated with a wide set of poses and sensor positions. The results of this work aims at providing the reader with details about the accuracy of the Kinect to help experimenters use this sensor in an optimal way under workplace conditions.

Section 2 provides details about the material and methods used to achieve this goal. Section 3 reports the results, *i.e.*, the accuracy of the reconstructed poses using a Kinect thanks to virtual mannequins. Results obtained with the virtual mannequin have been compared to actual measurements from a real subject for a limited set of poses in order to ensure that the virtual mannequin and the sensor simulation did not lead to unrealistic results. Section 4 discusses the result before a global conclusion.

## 2. Material and Methods

In this section, we describe the methods used to evaluate the accuracy of the Kinect measurements with numerical mannequins. One of the main difficulties is to accurately control, maintain, and repeat the poses performed by the subjects. Moreover it is difficult to accurately standardize the environment, which would also be captured by the sensor. Finally, designing experimental protocols to differentiate errors due to the sensor from those due to the pose estimation method is almost impossible. To overcome these limitations we propose to simulate numerical mannequins: (1) it provides us with accurate control of the poses and reliable ground truth data; (2) it enables us to focus on the errors caused by the pose estimation algorithm, assuming that the sensor is noise-free; (3) a given pose can be generated and tested several times to obtain more reliable statistical analysis; and (4) several sensor placements can be tested, which is complex to carry-out in real situations in an actual standardized manner for each tested human pose. Using a virtual mannequin instead of real subjects could introduce biases in the results, especially if the skinning of the character is not totally in accordance with a real human silhouette. Thus, to ensure that results obtained with the virtual mannequin could be used to estimate errors in real humans, we carried out an experiment with a real subject. The pose of the virtual mannequin is set to match its joint centers positions to those of the real subject to compare (1) the joint angles measured with the virtual mannequin and the simulated Kinect; (2) the actual joint angles of the skeleton; and (3) the joint angles provided by the actual Kinect sensor and software.

### 2.1. Method Overview

**Figure 1.**
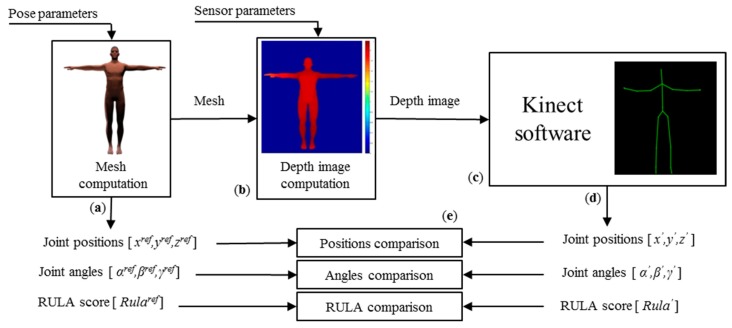
Overall method pipeline. (**a**) 3D meshes in specific poses, joint position values of reference (x^ref^, y^ref^, z^ref^ in meters), computed joint angle values of reference (α^ref^, β^ref^, γ^ref^ in degrees), and computed RULA score (Rula^ref^); (**b**) Depth images of the mesh in specific sensor positions; (**c**) Analysis of the depth images with the Kinect software for joint localization [15]; (**d**) Estimated joint position values (x′, y′, z′ in meters), computed estimated joint angle values (α′, β′, γ′ in degrees), and computed estimated RULA score (Rula′); (**e**) Measure error between values of reference and estimated values.

The pipeline of the method can be divided in three parts, as shown in Figure 1. In the first part, an anthropometric 3D mesh representing the surface of the human body is generated using MakeHuman software [31], as suggested in [30]. A skeleton is associated with the mesh to control the pose of this virtual human. As a consequence, changes in joint configuration leads to an adaptation of the 3D mesh thanks to linear blend skinning method available in the Blender software [32]. The resulting 3D mesh is exported in a standard *obj* file and the corresponding joint positions are exported in a separated text file.

In the second part, the behavior of the Kinect sensor is simulated by transforming the 3D mesh into depth images according to the imposed virtual sensor position and orientation in space, and the camera-intrinsic parameters. This way, we assume that the sensor is noise-free and is able to deliver accurate depth maps. The resulting 3D mesh is transformed into a depth map that is used as an input of the Kinect software. This software estimates the 3D position of the joint centers according to the Shotton's algorithm [15]. Let us consider now the method used to compare the results obtained with the Kinect software to the reference data (*i.e.*, the joint angles applied to the virtual mannequin).

### 2.2. Kinematic Parameters Used to Estimate the Accuracy of the Kinect Software

In this subsection, we describe the method used to compute joint angles according to the joint positions estimated by the Kinect software. First of all, one has to notice that if a pose sent to the Kinect is too different from the previous one, the system may need several frames to converge to a stable estimation. In this work we wish to send poses without any continuity in time and we consequently have to let the system converge to this stable solution. In the context of this experiment we have shown that the system converges after a maximum of 5 images. To take a security margin into account, we sent each pose to the Kinect software 15 times to guarantee reaching a stable estimation. In the end, only the final pose (the 15th one) is used in the following to analyze the accuracy of the system.

Once a stable pose is obtained, we have to compare the results to the reference values that were used to generate the 3D mesh of the virtual mannequin. The Kinect software only returns joint positions. An error in joint position would have a direct impact on the computation of joint angles and RULA score. Consequently, in this paper, we focused on three main parameters: joint position (shoulder, elbow, and wrist joint), joint angles (shoulder and elbow joint angles), and the corresponding RULA upper-limb score.

According to the estimated joint positions, joint angles should be computed using the ISB recommendation [33]. However the skeleton estimated by the Shotton's method is not compatible with this recommendation except for the humerus coordinate system. For the other joint coordinate systems, we have to proceed slightly differently according to the available points. For the trunk, the X-axis is defined as the normal of the plan formed by the spine joint (placed at the pelvis), the basis of the neck, and the right shoulder joint. The right shoulder joint is chosen to compute the trunk reference frame because it does not move significantly when performing left-arm motions. As the Kinect software does not deliver all the required kinematic information to compute the forearm reference frame according to the ISB recommendations, we alternatively use the vector convention detailed by [18].

Once the joint angles are known it is possible to compute RULA upper-limb scores [4]. For the shoulder joint, we need to isolate the flexion/extension angle and the abduction/adduction angles. The ISB guidelines propose to use three Eulerian angles to define the shoulder motion. The first one is the plane of elevation, which indicates if the elevation is an abduction (0°) or a forward flexion (90°). The second one is the elevation value of the shoulder. With these two Euler angles, we can compute the flexion and the abduction angles. As there is no available information for the wrist joint, the *wrist*, and *wrist twist* RULA scores are set the minimum values. Briefly, RULA is an assessment grid that consists of associating risks of musculoskeletal disorders to joint angles, repetition, and loads. A score is associated with joint angle intervals for each joint (such as 1 score for an elbow flexion in the [60°–100°] interval). Tables are used to compute a unique score (between 1 and 7) for each upper-limb and for the entire body taking repetition and loads into account. Hence RULA scores vary in a discontinuous manner between 1 and 7 according to continuous joint angles.

### 2.3. Protocol

**Figure 2.**
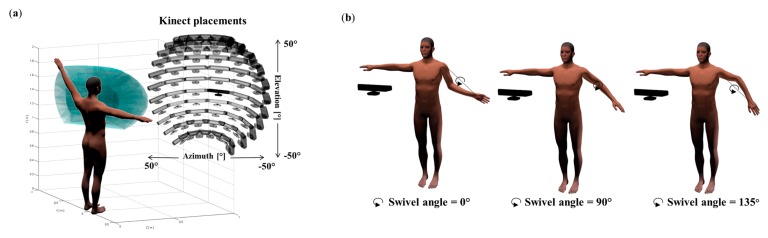
(**a**) Parameters of the experimental setup, the left hand reaching volume in blue (azimuth, elevation and elbow flexion) and the positions and orientations of the sensor in dark (azimuth and elevation); (**b**) The type of grip parameter: Grip from below at the left (0° swivel angle), grip from the side at the middle (90° swivel angle) and grip form above at the right (135° swivel angle).

In order to assess the accuracy of the Kinect in simulated workplace conditions, a large set of work poses (left-arm motions) in different sensor positions have been investigated. In this paper, we simulate poses limited to the reachable workspace frequently used in ergonomics [34]. Hence, Figure 2a shows that each pose can be defined by the relative position of the hand in the shoulder reference frame inside this reachable workspace. This position is defined by three parameters: azimuth (0° to 110° with a 10° step), elevation (−45° to 45° with a 10° step), and depth (associated with the elbow flexion ranging from 0° to 110° with a 10° step). If the hand position is fixed, the elbow is still free to swivel about a circular arc whose normal vector is parallel to the axis from the shoulder to the hand. Hence, we have chosen to sample the swivel angle in three main values: 0°, 90°, and 135° which corresponds to the main kinds of grips one can see in industrial work (see Figure 2b). Our parameters produce 4752 various poses. In this study, we have focused on the left elbow and shoulder joints, as the upper-limb is one of the most studied body parts in ergonomics (*i.e.*, in [35]).

Another important parameter is the placement of the Kinect as it is almost impossible to satisfy the constructor recommendation (placing the Kinect exactly 2 m in front of the subject) in actual industrial conditions. This is mainly due to occlusions in the workplace and the displacement of operators in this area. Thus the accuracy measured in perfect situation may underestimate the actual inaccuracy in workplace conditions. Consequently, we also tested the impact of the sensor's location on the accuracy of joint angle measurements. Thus, for each pose, the sensor position is defined relatively to the worker's position by two parameters: azimuth (−50° to 50° with a 10° step) and elevation (−50° to 50° with a 10° step), as described in Figure 2a. In this figure, the large set of Kinect positions is depicted in black. In each configuration, the virtual Kinect sensor is oriented to always look at the middle of the worker. 121 different sensor positions were consequently tested for each pose. Combining the number of poses with the number of sensor positions produces more than 500,000 configurations, which is impossible to evaluate in real situations.

For each of these configurations we compute the root mean square error (RMSE) between the resulting parameters (joint position, angle, and RULA score) and reference values applied to the virtual mannequin.

### 2.4. Comparison with Real Human

Using numerical mannequins to evaluate the accuracy of human limb motions is very interesting, as it enables us to control the test conditions with high precision for a huge set of configurations. However, it is natural to question if evaluations based on simplified numerical mannequins instead of real humans can lead to realistic results. To evaluate if the results obtained from the numerical mannequin are similar to those obtained from a real human, we carried out a complementary pilot study for a limited set of body configurations. We assume that similar joint angles in simulated and real measurements with an actual Kinect for a subset of configurations could help to ensure that the protocol based on the virtual mannequin is reliable.

**Figure 3.**
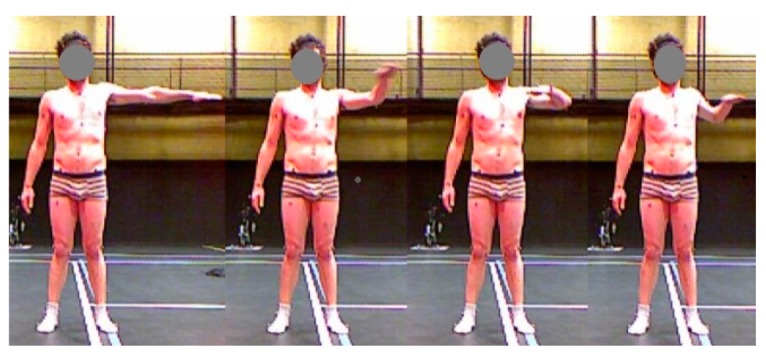
Examples of movement performed by the subject with 0° of elevation.

To this end, we asked a subject (age: 30, height: 175 cm, mass: 70 kg, right-handed) to perform a set of poses similar to those described above (*i.e.*, sampling the reachable space of the left arm). The subject was asked to make circles with his hand with three different elevations relative to the horizontal plane (−45°, 0°, 45°). These movements involve all the degrees of freedom of the left shoulder and elbow (see Figure 3). In this study, we only tested the configurations with a 90° swivel angle (see Figure 2b). Each circle for the given arm elevation was repeated five times.

Reflective markers were placed over standardized anatomical landmarks, in accordance with the International Society of Biomechanics recommendations [33]. The 3D positions of the reflective markers was collected with a 100 Hz Vicon-MX system (product of Oxford Metrics, Oxford, UK) composed of nine 4-Mpixels cameras. The Kinect sensor was placed 2 m in front of the subject, as recommended by [20]. The Kinect skeleton model was directly obtained at 30 Hz from the official Microsoft software development kit (Microsoft Kinect SDK v1.8) based on the Shotton's algorithm [15]. Let us denote the data provided by the Kinect as RK, and data provided by the Vicon motion capture system as MBS. The two systems were synchronized thanks to the inter-correlation method suggested in [36]. Data were also resampled to obtain the same timestamp to facilitate comparison. A fourth order low-pass Butterworth filter with a cut-off frequency of 6 Hz was applied on the angle data for both systems [18]. The root mean square error (RMSE) and the Pearson correlation between the three estimation methods is performed for the upper-limb joint angles.

Each reference pose measured on the subject with (MBS) was then retargeted to drive the numerical mannequin in a similar joint configuration using inverse kinematics, as reported in [37]. The position of the elbow and wrist joint centers were used as constraints of the inverse kinematics algorithm. This optimization method returns the joint angles minimizing the distance between virtual and real joint centers for the numerical mannequin.

**Figure 4.**
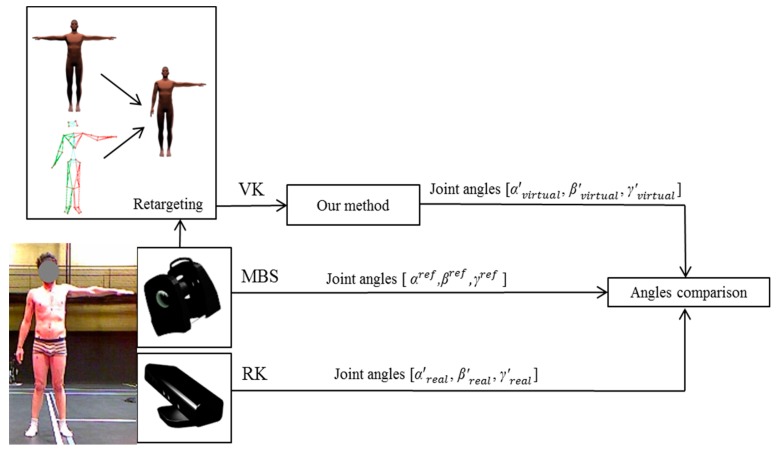
Experimental setup of the comparison of joint angles estimated with an optoelectronic motion capture system (MBS), the corresponding actual Kinect measurements (RK), and the simulated outputs using a virtual mannequin (VK).

Finally we applied the method described in Section 2.1 to compute expected outputs of the Kinect software based on the resulted virtual mannequin. Let us denote the data provided by this simulation of the Kinect sensor with the virtual mannequin as VK. The overall process is depicted in Figure 4.

## 3. Results

### 3.1. Evaluation of the Pose Estimation Accuracy for a Kinect Placed in Front of the Character

**Figure 5.**
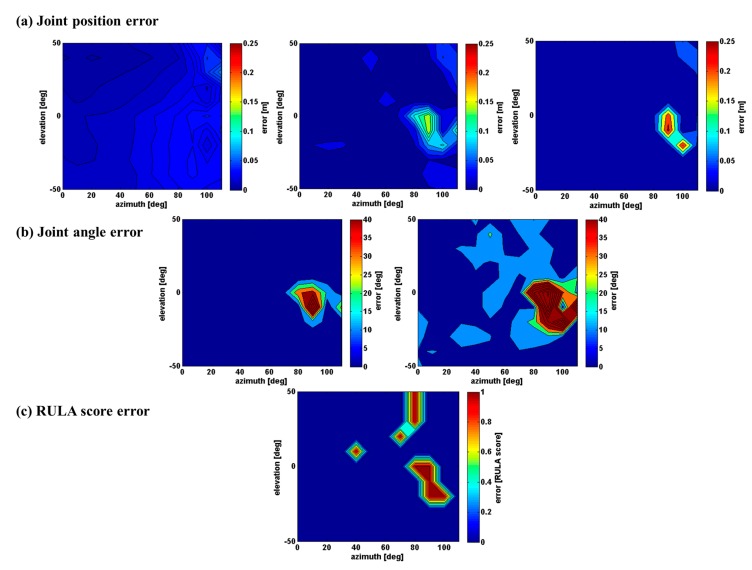
Accuracy of the Kinect measurement of the 135° swivel angle poses relative to azimuth and elevation pose parameters and with a zero elbow flexion (**a**) Error distribution of the shoulder (left), elbow (center) and wrist (right) joint positions estimated; (**b**) Error distribution of the shoulder (left) and elbow (right) joint angles calculated; (**c**) Error distribution of the resulting upper-body RULA score.

The first part of the results shows the accuracy of the estimated kinematic data when only the pose is changing, with a Kinect placed in front of the subject. Out of 4752 tested poses, we report the highlight of the results that seem representative of the global results (all the results are available as supplementary material: an executable program providing accuracy for all the possible upper-limb configurations). Figure 5 depicts the estimation error for the 135° swivel angle for all the azimuth and elevation configurations, with no elbow flexion. The first row depicts the error for the shoulder, elbow, and wrist joint positions. The second row depicts the error for the shoulder and elbow joint angles. The resulting RULA upper-body score is shown in the last row.

These results show that the accuracy of the shoulder joint position decreases with the growth of the azimuth pose parameter (Figure 5a left). However, the position error for the shoulder remains more stable than other joints (0.019 ± 0.009 m). For the elbow and wrist joint positions, a peak error is found in the same body configuration (azimuth near to 90° and elevation near to −10°) whereas there is no peak for the shoulder joint. The elbow and wrist errors increase to 0.16 m and 0.27 m respectively for this pose. For the rest of the pose configurations, the accuracy of the joint positions is acceptable; the overall average is 0.018 ± 0.023 m for the elbow joint and 0.024 ± 0.038 m for the wrist joint. As a consequence, the same peak error occurs for the shoulder and elbow angles (Figure 5b). The peak error is greater than 40° and the overall average error is 4.5° ± 8.9° and 12.6° ± 17.2° for the shoulder and elbow angles respectively. The same type of error is found for the RULA score in the last row of Figure 5c. Some other error peaks were measured for other body configurations, but they were due to limitations of the RULA score. It should be noted that RULA scores were attributed to ranges of angular values. Hence, two very close angular values near the boundaries of these ranges may lead to two different scores. For example, the peak error found at 40° of azimuth and 10° of elevation resulted from the reference shoulder flexion of 46°, which leads to a RULA score of 3, while the estimated shoulder flexion is 44° leads to a score of 2.

**Figure 6.**
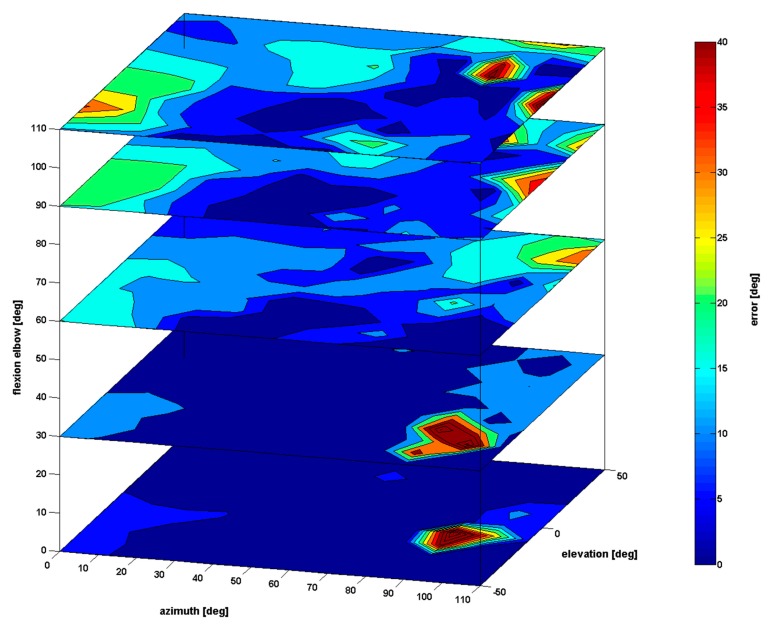
Accuracy of the Kinect measurement of the shoulder angle (0° swivel angle) according to three parameters: azimuth, elevation, and elbow flexion.

Figure 6 depicts the evolution of the shoulder angle error according to three parameters: azimuth, elevation, and elbow flexion. The error increases with the elbow flexion parameter for an azimuth values close to 0° and an elevation value close to −50°. The error peak measured in Figure 5b also appears when elbow flexion is null (azimuth close to 90° and elevation close to −10°). However, this error area disappears when the elbow flexion increases. For little elbow flexion (bottom part of Figure 6), except for this particular area, the shoulder angle is accurately estimated (less than 8°). On the contrary, for high azimuth values (close to 110°), elevation (close to 50°), and elbow flexion (close to 110°), inaccuracy remains significant (values up to 51°). Finally the estimation error of the shoulder angle is more important when the elbow flexion parameter increases in the left part of the Figure 6 (azimuth close to 0° and elevation close to −50°).

**Figure 7.**
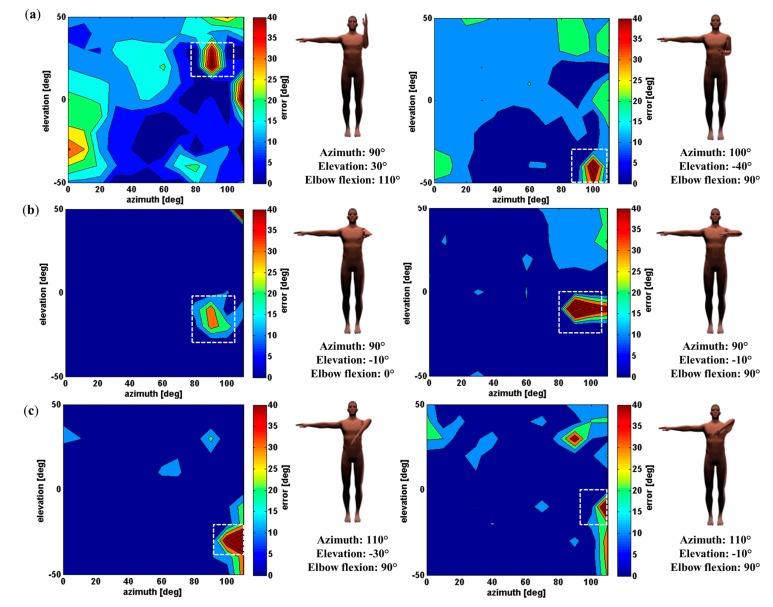
Example of misestimated poses with a Kinect placed in front of the subject for the shoulder joint (left column) and the elbow joint (right column). Dotted squares show the graphic areas selected. (**a**) Swivel angle at 0°; (**b**) Swivel angle at 90°; (**c**) Swivel angle at 135°.

Figure 7 illustrates several examples of misestimated poses for a Kinect placed in front of the mannequin. For each selected pose, the figure depicts its error map and the corresponding mesh. Each row depicts a pose associated with a specific swivel angle (a: 0°, b: 90°, and c: 135°). The left column of this figure depicts the shoulder joint angle error and the right column depicts the elbow joint angle error. For this figure, only poses with error values greater than 30° were selected. Pose estimation dropouts are clearly visible for all the examples and are highlighted with dotted square.

### 3.2. Evaluation of the Pose Estimation Accuracy for Various Kinect Placements

**Figure 8.**
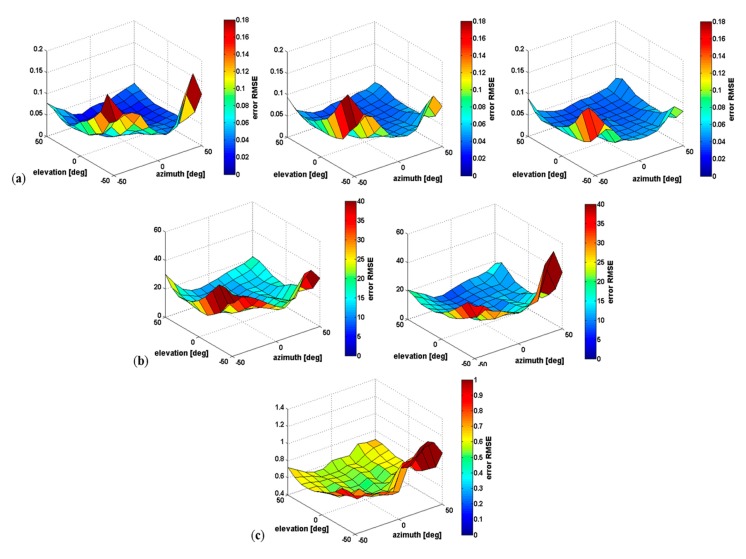
Root mean square error (RMSE) of all poses relative to the sensor placement (azimuth and elevation). (**a**) RMSE distribution of the shoulder (left), elbow (center), and wrist (right) joint positions estimated; (**b**) RMSE distribution of the shoulder (left) and elbow (right) joint angles calculated; (**c**) RMSE distribution of the resulting upper-body RULA score.

The second part of the results shows the impact of the Kinect placement relative to the subject on pose estimation accuracy. The RMSE is computed for all the poses relative to the azimuth and elevation of the sensor placement (see Figure 8). Row (a) depicts the RMSE distribution of the estimated joint positions of the shoulder, elbow, and wrist. Row (b) illustrates the RMSE for the shoulder and elbow joint angles. Finally the impact of the error on the upper-body RULA score is shown in Row (c). First of all, results show that the RMSE is more important when the sensor is placed at a low position (sensor elevation = −50°), especially for large azimuth values. Indeed, the sensor placed on the opposite side of the arm measured (sensor azimuth = −50°) leads to misestimation of the shoulder, elbow, and wrist joint positions (Figure 8a), up to 0.18 m, 0.18 m, and 0.15 m respectively. However, one can notice that when the sensor is placed on the same side as the measured arm (sensor azimuth = 50°) it leads to a significant RMSE for the shoulder position (Figure 8a left), up to 0.17 m. This shoulder position error consequently leads to high errors in joint angles (shoulder and elbow errors up to 56° and 41° respectively) and RULA score up to 1.16 (Figure 8b,c).

### 3.3. Comparison to Results Obtained with Real Human

Results are summarized in Table 1. When comparing motion capture data (MBS) to real (RK) and virtual Kinect (VK), one can see that error with the virtual Kinect is lower than those of the real Kinect. This is compatible with the fact that the virtual Kinect is noise-free compared to the real one, leading to lower error. For the shoulder elevation angle, comparison between RK and VK exhibits an 8.5° (±3.6) RMSE, and a Pearson correlation of *p* = 0.86. For the elbow joint angle, comparison between RK and VK exhibits a 13° (±2.3) RMSE, and a Pearson correlation of *p* = 0.96.

The results of this pilot study tend to show that our evaluation method based on a virtual mannequin exhibit similar results than when using a real Kinect.

## 4. Discussion

In this study, we have estimated the accuracy of the Kinect in simulated workplace conditions for a large set of work poses and with different sensor positions. This paper aims at providing the reader with details about the accuracy of the Kinect software to provide optimal methods for its use in workplace conditions. Our results are in accordance with previous works, especially for the accuracy of the estimated joint position [23,24] and joint angles [24,25]. It remains difficult to compare our work with others because most of them generally measure planar gestures in 2D anatomical plans. Previous studies were also limited to a Kinect placed in front of the subject, and the impact of the device position relative to the subject was generally not evaluated. Only one work measured the accuracy of the Kinect for different orientations of the subject, ranging from the frontal to the side view [23]. However, only four orientations were tested, which is insufficient to evaluate the impact of the device position relative to the subject. As the method presented in this paper uses virtual mannequins to simulate the depth images, on can consider that the evaluation method is independent of the hardware device and focuses on the error about the pose estimation method.

The first part of the results showed that the pose estimation algorithm was acceptable for most applications (for example the shoulder position error is 0.025 m), except for a few specific body configurations. For these poses, the error can grow up to more than 40° for the shoulder joint angle (see Figures 5, 6 and 7). In these cases, some body parts are partially occluded by other body segments in the Kinect axis (Figure 7) and it is almost impossible for the Kinect to correctly perceive the arm and the forearm. For example, in Figure 7b left, the error of the shoulder joint angle is high when the arm is straight and aligned with the Kinect axis. In this body configuration the segments are not well measured and the estimation of the joint center location is not accurate.

In the second part of the results, we find that that the sensor position could have an important impact on the pose estimation accuracy. Specifically, the elevation of the Kinect has a greater impact compared to azimuth (Figure 8). Indeed, a Kinect placed at a low position (sensor elevation = −50°) leads to a large error of the pose estimation (up to 0.169 m for the shoulder position error), especially for large azimuth values (sensor azimuth = −50° and 50°). This particular sensor placement lead to partial occlusions of body parts of interest. In other cases, the kinematic information provided by the Kinect software is acceptable for most applications (0.052 ± 0.03 m for the shoulder position error). One has to consider that results could be worse than those reported in this paper when using a real Kinect because of noise that was not taken into account here.

In the third part of the results, the measured joint angles of a real human were correlated with those obtained with a real Kinect and method simulated Kinect based on the virtual mannequin. In this pilot study, the errors reported when using our method were similar to those obtained with a real Kinect. However, one can notice that the results obtained with the simulated Kinect were slightly better than those obtained with the real Kinect, which is in accordance to the fact that the virtual sensor is noise-free while the actual sensor is noisy.

The results of this study highlight the importance of the design of the motion capture protocol with a Kinect. Unlike other works, this study aims to evaluate both complex poses and various sensor placements. On one hand, these situations are far from ideal conditions for Kinect measurements. On the other hand, they tend to mimic natural situations encountered in actual workplaces. The results presented in this paper can help experimenters to resolve constraints imposed by these ecological situations.

In this paper almost 500,000 configurations (body pose and Kinect position) have been tested. Thus, this evaluation method provides a huge amount of data, which makes it difficult to present in a compact manner in this contribution. We have chosen to highlight some of the extreme values and an overall average error value.

In this study, the RULA evaluation is limited to the shoulder and elbow joints. It will be interesting to measure the other body parts requested by the RULA method (trunk, neck, wrist, and leg), to quantify their impacts on the resulting RULA score. To this end it would be necessary to apply the same method to these joint angles in a new protocol.

The Kinect software does not deliver all the required kinematic information to compute the reference frames according to the ISB recommendations. In this work we slightly adapted the trunk reference frame to compute the joint angles of the shoulder. We also used an alternative computation for the elbow joint, as proposed by [18]. This lack of information would be a problem if we implement a biomechanical model from the Kinect data, such as using inverse dynamics to estimate joint torques and forces. To tackle this problem, [38] developed a model-based approach to enhance the anatomical accuracy of the standard skeleton provided by the Kinect. It could also be applied to the method developed in this paper in order to satisfy the ISB recommendations.

The method presented in this paper relies on a virtual mannequin, which strongly influenced the results. Hence the shape and the anatomical model of this mannequin could be slightly different from that of a real human, and thus could drive the evaluation to inappropriate results. In the future, it would be essential to evaluate the sensibility of the evaluation method to the choice of the mannequin. For example, we could compare the results obtained with a large set of virtual mannequins. Moreover, it would be interesting to compare results obtained from a real human with those obtained from the virtual mannequin, with a larger set of subjects.

This method allowed us to evaluate specific poses and sensor configurations in an accurate, reliable, and standardized manner, even if the intrinsic noise of the device were not considered here. The process is fully automatic. It enables an experimenter to quickly perform an evaluation. In addition, it is also possible to simulate occlusions that could occur in real situations by adding virtual objects in the environment. For example, we could add clothing to the virtual mannequin, or a specific workstation in front of him.

## 5. Conclusions

The study presented in this work suggests that the Kinect software can be a useful motion capture tool for ergonomic evaluation. In most of the results reported in this work, the accuracy is enough to correctly fill-in ergonomic assessment grids like the RULA grid. However large errors can occur in specific cases that have been reported in this paper, such as when the arm is aligned with the Kinect sensor. We also noticed that sensor placement may lead to bad estimations. Hence, an experimenter has to carefully design his protocol according to these results. He has to deal with constraints of the environment (such as occlusions or possible sensor placements) while taking these recommendations into account.

The results reported in this study have been obtained with a new systematic method based on a virtual mannequin. It allows us to automatically estimate the accuracy of the system for numerous complex poses and sensor positions, which would not be possible with classical methods and protocols. Moreover, this method focuses on the accuracy of the pose estimation algorithm provided by the official Kinect software. It assumes that the device is noise-free and delivers perfect depth images. Hence results with an actual Kinect sensor would certainly be worse than those reported in this paper. However, we assumed that being able to separate errors due to the sensor from those due to the method is a strong advantage, even for future upgrades of the measurement systems, such as the new Kinect 2. It would be possible to add noise to the simulated depth images used as an input in this work to simulate potential noise of the sensor. However, in this paper, we reported numerous results about the impact of experimental setups on the skeleton reconstruction, assuming perfect 3D data provided by the Kinect. Dealing with depth images inaccuracies would again increase the complexity of the results reported in this paper. In the future, it would be interesting to develop an online test bench based on the virtual mannequin and sensors. It would help experimenters to test the preliminary expected accuracy for their recordings according to the experimental conditions. It would also enable us to extend this accuracy analysis to the entire body.

From the ergonomic point of view, correctly using a Kinect in actual workplace conditions would enable ergonomists to analyze motions instead of isolated poses. It provides supplementary temporal information, such as the time spent above a given RULA score. It could also be used as real-time feedbacks, as suggested in [39].

## Figures and Tables

**Table 1.** Root mean square error (*RMSE*) is expressed in degree (st. dev.), *p* = Pearson coefficient of correlation.

		**MBS-RK**	**MBS-VK**	**RK-VK**
Shoulder joint angle	*RMSE*	9.5° (±2.9)	5.2° (±1.5)	8.5° (±3.6)
	*p*	0.83	0.92	0.86

Elbow joint angle	*RMSE*	11.4° (±2.0)	8.2° (±1.3)	13.0° (±2.3)
	*p*	0.97	0.98	0.96
